# Shoulder function and work disability after decompression surgery for subacromial impingement syndrome: a randomised controlled trial of physiotherapy exercises and occupational medical assistance

**DOI:** 10.1186/1471-2474-15-215

**Published:** 2014-06-21

**Authors:** Susanne W Svendsen, David H Christiansen, Jens Peder Haahr, Linda C Andrea, Poul Frost

**Affiliations:** 1 Department of Occupational Medicine, Danish Ramazzini Centre, Regional Hospital West Jutland - University Research Clinic, Herning, Denmark; 2 Department of Occupational Medicine, Danish Ramazzini Centre, Aarhus University Hospital, Aarhus, Denmark

**Keywords:** Acromioplasty, Oxford Shoulder Score, Physiotherapy, Randomised controlled trial, Rehabilitation, Shoulder, Sickness absence, Surgery, Training, Work retention

## Abstract

**Background:**

Surgery for subacromial impingement syndrome is often performed in working age and postoperative physiotherapy exercises are widely used to help restore function. A recent Danish study showed that 10% of a nationwide cohort of patients retired prematurely within two years after surgery. Few studies have compared effects of different postoperative exercise programmes on shoulder function, and no studies have evaluated workplace-oriented interventions to reduce postoperative work disability. This study aims to evaluate the effectiveness of physiotherapy exercises and occupational medical assistance compared with usual care in improving shoulder function and reducing postoperative work disability after arthroscopic subacromial decompression.

**Methods/Design:**

The study is a mainly pragmatic multicentre randomised controlled trial. The trial is embedded in a cohort study of shoulder patients referred to public departments of orthopaedic surgery in Central Denmark Region. Patients aged ≥18–≤63 years, who still have shoulder symptoms 8–12 weeks after surgery, constitute the study population. Around 130 participants are allocated to: 1) physiotherapy exercises, 2) occupational medical assistance, 3) physiotherapy exercises and occupational medical assistance, and 4) usual care. Intervention manuals allow individual tailoring. Primary outcome measures include Oxford Shoulder Score and sickness absence due to symptoms from the operated shoulder. Randomisation is computerised with allocation concealment by randomly permuted block sizes. Statistical analyses will primarily be performed according to the intention-to-treat principle.

**Discussion:**

The paper presents the rationale, design, methods, and operational aspects of the Shoulder Intervention Project (SIP). SIP evaluates a new rehabilitation approach, where physiotherapy and occupational interventions are provided in continuity of surgical episodes of care. If successful, the project may serve as a model for rehabilitation of surgical shoulder patients.

**Trial registration:**

Current Controlled Trials ISRCTN55768749.

## Background

Subacromial impingement syndrome (SIS) is an important cause of ill-health with a prevalence of 2-8% of the working population [1,2]. When non-surgical treatment fails, surgery may be chosen [3,4], and most often subacromial decompression is performed. Substantial increases in rates of surgical treatment of SIS have been reported from Sweden [5], the US [6], the UK [7], and Denmark [8]. In Denmark, a level of 0.15% of the working age population was reached in 2008. Although high proportions with successful outcomes have been reported, around 20% of the patients experience chronic shoulder pain and/or disability after surgery [9,10]. In Denmark, 10% of employed patients leave the labour market within two years after surgery due to health related disability [8].

Considering the fact that exercise therapy is widely used to help restore shoulder function after surgery (Christiansen et al., submitted, [11,12]), few randomised controlled trials (RCTs) have compared effects of different postoperative exercise programmes [13-16]. For musculoskeletal disorders, current evidence indicates that workplace-oriented interventions may be effective to promote work retention [17,18]. The majority of studies have focussed on low back pain [17,18], but different disease-specific risk profiles indicate that low back pain and upper extremity disorders may need different interventions [19]. Although work disability may lead to a poorer quality of life and loss of social identity [20], usual care of surgical shoulder patients does not focus on job retention as an important outcome, and to our knowledge, workplace-oriented interventions to promote work retention after subacromial decompression for SIS have not been evaluated.

The hypotheses of the Shoulder Intervention Project (SIP) are that postoperative physiotherapy exercises are more effective than usual care in improving shoulder function and that postoperative occupational medical assistance is more effective than usual care in promoting work retention.

## Methods/Design

### Design and setting

**Figure 1:**
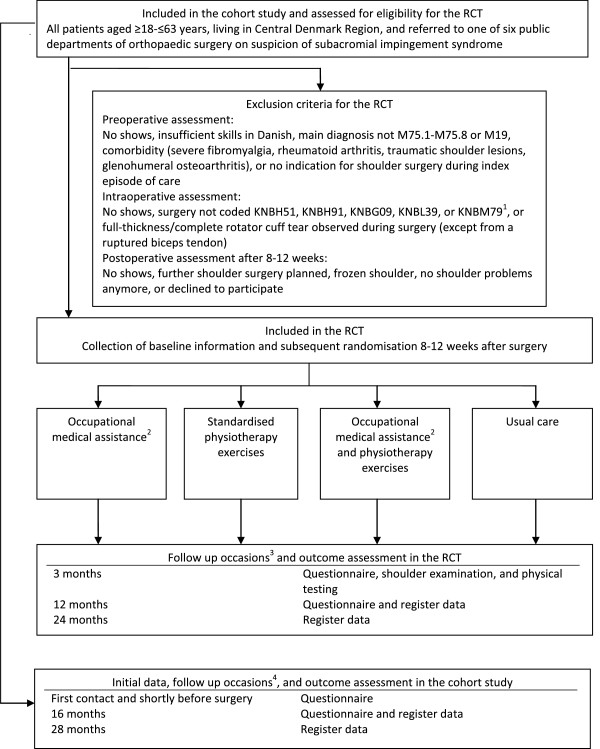
**Flow chart following patients from inclusion into the cohort study through each stage of the randomised controlled trial to final data collection.** ^1^The Danish version of the Nordic Medico-Statistical Committee (NOMESCO) Classification of Surgical Procedures; ^2^Only patients in paid work for ≥25 hours per week; ^3^T_0_ = baseline; ^4^T_0_ = first contact to a department of orthopaedic surgery after inclusion into the cohort study.

The study is a mainly pragmatic multicentre RCT [21]. The RCT is embedded in a cohort study, which comprises all patients aged ≥18–≤63 years who are referred to one of six public departments of orthopaedic surgery in Central Denmark Region on suspicion of SIS in a three year period, 2011–2014. Figure 1 presents the inclusion of patients and the stages of the RCT. The physiotherapy intervention will be evaluated for patients with and without paid work. The occupational intervention (and the physiotherapy intervention) will be evaluated for the subgroup of patients, who have paid work for at least 25 hours per week, using a factorial design [22,23]. Table 1 presents variables in the RCT and in the cohort study, in which the RCT is embedded.

**Table 1:** Overview of variables in the randomised controlled trial (RCT) and in the cohort study, in which it is embedded

**Variable**	**Time of data collection and population**						
	**First orthopaedic contact**	**Shortly before surgery**	**Baseline**	**3 months**	**12 months**	**16 months**	**24/28 months**
	**Cohort**	**Cohort**	**RCT**	**RCT**	**RCT**	**Cohort§**	**RCT/Cohort§**
**Questionnaire**							
Job title	x	x	x	x	x	x	
Psychosocial working environment			x				
Work ability	x	x	x	x	x	x	
Work productivity and activity impairment				x	x		
Work modifications				x			
Advice to stop working				x			
Job centre initiated workplace visits				x			
Compensation claims	x		x				
Oxford Shoulder Score	x	x	x	x	x	x	
Shoulder pain intensity			x	x	x		
Duration of shoulder symptoms	x	x					
Global impression of change			x (since shortly before surgery)	x (since baseline)	x (since assessment at 3 months)		
Other musculoskeletal complaints		x	x		x		
EQ-5D-3L*	x	x	x	x	x	x	
Use of analgesics		x	x	x	x		
Non-surgical treatment		x (previous 3 months)	x (since operation)	x (previous 3 months)	x (previous 3 months)	x (previous 3 months)	
Expectations to surgery		x			x (fulfilment)		
Patient preferences			x				
Height, weight, handedness		x					
Smoking		x					
Physical activity		x	x	x	x		
Fear avoidance beliefs			x	x	x		
Self efficacy			x				
Mental health		x	x	x	x		
Satisfaction with SIP*				x			
Shoulder-related sickness absence				x			
Full return to own or other work with equal earnings					x	x	
**Shoulder examination and physical testing**							
Constant Score			x	x			
Range of motions			x	x			
Jobe’s test			x	x			
Painful arc test			x	x			
Hawkins’ test			x	x			
Scapular dyskinesis			x	x			
Maximum oxygen uptake			x	x			
Height, weight			x	x			
**Register data**							
Number of weeks receiving health-related transfer incomes					x	x	x

^§^At 16 and 28 months after first contact to a department of orthopaedic surgery, data is collected for the cohort except for patients in the RCT, for whom corresponding and more detailed data is collected at 12 and 24 months after RCT baseline. *Abbreviations are explained at the end of the paper.Regarding follow up occasions, please refer to Figure 1.

### Participants

To be included in the RCT, patients must have surgery under a main diagnosis of SIS or acromioclavicular osteoarthritis (International Classification of Diseases 10^th^ revision: M75.1-M75.8 or M19) and with a main shoulder surgery code of arthroscopic subacromial decompression (KNBH51, KNBH91, KNBG09, KNBL39, or KNBM79 according to the Danish version of the Nordic Medico-Statistical Committee Classification of Surgical Procedures). Exclusion criteria are shown in Figure 1. At postoperative clinical control after 8–12 weeks, eligible patients are provided with information material on SIP and are asked to participate in a brief telephone interview. Patients are only recruited if they have at least slight shoulder problems doing usual activities (e.g. work, study, housework, family or leisure activities) when assessed on a five level scale (no problems, slight problems, moderate problems, severe problems, unable). Furthermore, the patients’ employment status is assessed. Patients can be randomised to one of all four arms of the RCT if they are in paid work for at least 25 hours per week. Remaining patients can only be randomised to one of the two arms, which do not include occupational medical assistance. To be included, patients must also consent for acquisition of medical records. Figure 1 presents the inclusion procedure, which has been effective since April 2012, when it was changed due to slow recruitment. Until then, patients were excluded in case of previous shoulder surgery and/or diabetes, if they were not in paid work for at least 25 hours per week, and if they were not fulltime sick-listed.

Participation in SIP is based on the principles of informed consent. Participants are covered by the Danish patient insurance system. SIP provides compensation for documented loss of earnings and transportation costs. The study has been approved by the Central Denmark Region Committees on Biomedical Research Ethics (identification number: M-20100131) and by the Danish Data Protection Agency (journal number: 2010-41-4316).

### Interventions

#### Occupational medical assistance

After randomisation, the patient is seen by an occupational physician from the research team, who assesses the patient’s work instability using a gold standard approach [24]. Work instability is characterised by a mismatch between an individual’s functional capabilities (in this case shoulder function) and job demands (in this case biomechanical shoulder load) to an extent where job retention is threatened [24]. Shoulder function is assessed by clinical examination and shoulder load is assessed using a job exposure matrix (JEM), both combined with a semi-structured interview. Depending on the degree of work instability, a three month action plan is constructed. The plan is attuned to the patient’s most important barriers against continuing or resuming work, in agreement with the biopsychosocial model [25,26].

A JEM based on expert ratings is used [27]. The JEM comprises shoulder force requirements using a 5-point force score scale, postural load in terms of daily duration of work with the arm elevated >90°, daily duration of moderately repetitive work with ≥4-<15 upper arm movements per minute, and highly repetitive work with ≥15 upper arm movements per minute. Jobs are classified as having *high shoulder load* (indicated by a red colour code) if they fulfil at least one of the following criteria: a force-score ≥3, upper arm elevation >90° ≥1 hour/day, highly repetitive work ≥½ hour/day, and moderately repetitive work ≥4 hours/day. Remaining jobs are classified as having *medium shoulder load* (indicated by a yellow colour code) in case of highly repetitive work <½ hour/day and at least one of the following: a force-score >1.5-<3, upper arm elevation >90° ≥½-<1 hour/day, and moderately repetitive work ≥2-<4 hours/day. Those jobs, which fulfil all of the following criteria, are classified as having *low shoulder load* (indicated by a green colour code): a force-score ≤1.5, upper arm elevation >90° <½ hour/day, highly repetitive work <½ hour/day, and moderately repetitive work <2 hours/day. In the semi-structured interview, the physician gets a description of the patient’s primary work tasks in order to individualise the JEM-based exposure assessment and to identify work tasks with relatively high shoulder load. The interview also covers shoulder symptoms, general health status, and the patient’s assessment of the most important barriers against continuing or resuming work. The physician interprets and scores the patient’s 1) shoulder function, 2) shoulder load, 3) worries that the work may harm the shoulder, even though the job entails low shoulder load, 4) influence on the way work tasks are performed and on task distribution, and 5) support from employer/supervisor or colleagues with respect to work modifications. Each of these five items is scored on an 11-point numeric rating scale ranging from 0 (no problem) to 10 (the largest possible problem).

Table 2 presents our algorithm to assess work instability, indications for workplace visits, and intervention levels. An indicated workplace visit may be omitted if the patient can arrange workplace adaptations him-/herself, or if the employer is against a visit. In some cases, it may suffice that the physician contacts the workplace by telephone. Workplace visits are performed within 10 working days, attended by the occupational physician, the patient, and the employer/supervisor. The duration is around one hour. The physician assesses the patient’s shoulder load by observation. Tasks with relatively high shoulder load are identified, and potential solutions are discussed in order to reach an agreement on work adaptations that are feasible within a short time horizon, i.e. adaptations characterised by low costs, low complexity, and compatibility with existing work structures. Deadlines are set for implementation of the adaptations and their duration is stipulated. Advice on far-reaching, long-term adaptations may be passed on. The physician classifies the planned workplace adaptations as technical solutions, reductions of working hours (part-time sick-listing), or modifications of task distribution [28]. The plan may represent more than one of these categories. To enable coordination of workplace-oriented efforts, the physician may contact the patient’s municipal job centre.

**Table 2:** **Algorithm to assess work instability, indications for workplace visits, and intervention levels, modified from **[24]

**Level of work instability**	**Specification of work instability**	**Indication for workplace visit and level of intervention**
Level 0	Shoulder function is adequate to perform all work activities, pain is under control, work activities do not imply a risk to the shoulder (job colour code: green), and the patient does not worry that this is the case.	No
Level 1	As above, but from time to time pain is a problem, work activities do not imply a risk to the shoulder (job colour code: green), but the patient worries that this may be the case, and/or the patient experiences that the employer hesitates to let the patient do his or her ordinary work activities in order to protect the shoulder.	Maybe – the indication is relative. Reassure the patient (and the employer) that work can be continued/resumed.
Level 2	Work activities do not imply a risk to the shoulder (job colour code: green), but pain is aggravated to an unacceptable level, and/or shoulder function does not match all work activities. The shoulder problems are expected to resolve within 6–12 months.	Yes, temporary solutions have to be established at the workplace.
Level 3	Some work activities imply a risk of worsening the shoulder condition (job colour code: yellow or red), pain is aggravated to an unacceptable level, and/or shoulder function does not match all work activities. The shoulder problems are not expected to resolve within 6–12 months.	Yes, permanent solutions have to be established at the workplace.
Level 4	Major work activities imply a risk of worsening the shoulder condition (job colour code: yellow or red), pain is aggravated to an unacceptable level, and/or shoulder function does not match the work demands. The shoulder problems are not expected to resolve within twelve months.	Yes, a permanent shift to another job may be necessary.
Undetermined	A workplace visit is necessary to assess work instability.	Yes, shoulder load has to be assessed.

Job colour codes (middle column) are based on a job exposure matrix combined with a semi-structured interview.

The patient receives a note with the agreed plan, advice on general physical activity, and transference of questions concerning analgesics to the patient’s general practitioner. If warranted, the physician notifies the shoulder disorder as a possible industrial injury in accordance with Danish legislation.

After six weeks, the physician contacts the patient by telephone to assess adherence to the plan. If needed, the employer/supervisor is contacted. Three months after baseline, the patient is seen for final evaluation and workplace-oriented advice. The patient’s assessment of factors facilitating or hindering the plan is registered, and any adverse events are noted. The intervention is described in a detailed manual.

#### Physiotherapy exercises

A standardised physiotherapy exercise intervention has been developed based on a systematic literature review and meetings with clinical physiotherapists working in the field to combine evidence and practical experience. The process of developing the intervention and the intervention itself are presented in a separate publication (Christiansen et al., in prep). The programme is described in a detailed manual that presents the exercises, the number of repetitions at each training level, and criteria for progression.

Depending on their need for supervision, the patients attend a physiotherapist at a municipal training centre 8–15 times within a period of eight weeks, including an initial and a final clinical evaluation. The physiotherapist-supervised individual training sessions last up to 60 minutes each. The patients are instructed to perform additional self-training. At baseline, advice is given on general physical activity, questions concerning analgesics are transferred to the patient’s general practitioner, and the patient is advised to refrain from other specific shoulder training during the intervention period. The patient keeps a self-training diary, and the physiotherapist registers any deviations from the manual and scores patient adherence. Any adverse events are noted.

#### Occupational medical assistance and physiotherapy exercises

This entails a combination of the two interventions described above.

### Usual care and co-interventions

At three month follow up, questionnaire information is collected about non-surgical treatment in terms of number of treatments by physiotherapists, physicians, chiropractors, and other health care providers, types of treatment (shoulder training, subacromial injections, manual therapy, and shock wave therapy), use of analgesics, work modifications due to the operated shoulder, job centre initiated workplace visits, and any advice to stop working or find other employment given from whom, see Table 1.

### Primary outcome measures

To evaluate the physiotherapy intervention at three months, the primary outcome measure will be change in shoulder function since baseline assessed by *Oxford Shoulder Score* (OSS), which ranges from 0 to 48 with 48 being the best outcome [29]. To evaluate the occupational intervention at three months, the primary outcome measure will be the *sickness absence percentage*, i.e. the number of hours of sick-leave due to symptoms from the operated shoulder in relation to the number of planned working hours within three months from baseline. This information is gathered by one month day-by-day calendars. Any hours off work to participate in the project will count in the outcome measure.

To evaluate the physiotherapy intervention at 12 months, the primary outcome measure will be *OSS.* For the subgroup, who are in paid work for at least 25 hours per week, the occupational intervention will be evaluated at 12 and 24 months, and the physiotherapy intervention will be evaluated at 24 months, using the primary outcome measure *transfer income percentage*, i.e. the number of weeks receiving temporary or permanent health-related transfer incomes within 12 and 13–24 months from baseline, respectively, divided by 52 weeks, according to the Danish National Register on Public Transfer Payments [30].

As a consequence of the change of inclusion criteria, we had to change the intended primary outcome measures for the occupational intervention at 3 and 12 months. The original measures at these follow up occasions were duration of fulltime sick-leave and duration of sick-leave until lasting return to work, respectively.

### Secondary and supplementary outcome measures

At three months, *Constant Score* will be used as a secondary outcome measure for the physiotherapy intervention [31-33], and *OSS* will be used as a secondary outcome measure for the occupational intervention to evaluate any deterioration. For both interventions, the *Fear Avoidance Belief Score*[34], modified to focus on the shoulder [35] will also be used. Furthermore, the physiotherapy intervention will be evaluated using the *sickness absence percentage* for the subgroup in paid work for at least 25 hours per week.

At 12 months, the *Fear Avoidance Belief Score*, modified to focus on the shoulder, will be used as a secondary outcome measure for both interventions, and *OSS* will be used as a secondary outcome measure for the occupational intervention. For the subgroup, who are in paid work for at least 25 hours per week, the physiotherapy intervention will be further evaluated using the *transfer income percentage.*

For both interventions, supplementary outcome measures will be chosen from the variables presented in Table 1. As an annex to the RCT, participants are asked to answer a short text message once a week for 12 weeks after baseline, rating their pain at rest within the last 24 hours, using an 11-point Numeric Pain Rating Scale ranging from 0 (no pain) to 10 (worst imaginable pain).

### Randomisation

At the region’s two departments of occupational medicine, participants are individually randomised by a research secretary, using a computerised random number generator with stratification by surgical department and blocking within strata using randomly permuted block sizes of 12, 8, and 4. Blocking within strata is used to ensure an equal distribution of the interventions between the surgical departments. Randomly permuted block sizes ensure allocation concealment. Once performed, the randomisation result is automatically locked by SIP-online (see below).

### Data collection and blinding

All project activities are documented in a web-based data management system, SIP-online (Trial Partner®), tailored to the project. SIP-online is password protected, and data is transferred via secure lines (https) to a server protected by firewalls. Data is backed up every 24 hours. After closure of the project, data will be stored at the Danish Data Archives.

Before randomisation and at 3 month follow up, shoulder examination and physical testing is performed by a research physiotherapist. At 3 months, blinding of the research physiotherapist for group assignment is ensured by restricted access to SIP-online and instructions to the participants not to tell him about their group assignment. The success of blinding of the outcome assessor will be evaluated.

### Process evaluation

The degree to which the project reaches the targeted group of patients will be assessed by comparing the RCT group with other patients from the cohort with respect to *OSS*, *full return to own or other work with equal earnings*, and *transfer income percentage* within specified time periods after surgery.

For the occupational intervention, process evaluation will include the timeline of the process as compared to the protocol, the number of workplace visits in relation to the number indicated, and the degree to which planned workplace adaptations are implemented (a score from 0 to 10). For the physiotherapy intervention, adherence to the protocol will be evaluated in terms of the percentage of participants where the minimum number of supervised training sessions is completed, the percentage of participants where deviations from the training protocol are registered, and the percentage of participants who – according to their training diary - perform self-training with the advised frequency.

Participant preferences with respect to intervention group [36], and satisfaction with participation in SIP are covered by questionnaires at baseline and three month follow up, respectively, as shown in Table 1.

### Statistical analyses

Trial results will be reported as a summary of the outcome measures in each group together with the estimated effect size and its precision. Statistical analyses will be performed according to the intention-to-treat principle, which will be supplemented by per protocol analyses depending on the proportion who do not receive the intended treatment. For outcomes measured using an essentially continuous scale (e.g. *OSS, sickness absence percentage,* and *transfer income percentage*), differences between groups at follow up will be compared by means of linear regression modelling with appropriate transformation of dependent variables. Analyses of effects of the physiotherapy intervention will be adjusted for centre (department of occupational medicine), for the occupational intervention (no, yes, irrelevant), and for the outcome measure at baseline. For the subgroup, who are in paid work for at least 25 hours per week, a two-by-two factorial design will be used. The analyses will focus on main effects of the occupational intervention, but cell-by-cell results will also be reported [22,23]; the analyses will be adjusted for centre. Numbers needed to treat for one patient to benefit from the interventions will be calculated [37]. In addition to main RCT results, supplementary analyses will be performed using variables presented in Table 1.

### Sample size calculations

The calculations are based on the primary outcomes *OSS, sickness absence percentage, and transfer income percentage.* With 65 participants, who receive physiotherapy exercises, and 65 participants, who do not, a difference in mean *OSS* of 2.4 points can be detected at 12 months with power = 0.8, alpha = 0.05, an assumed standard deviation of 9.0 *OSS* points, a correlation between *OSS* at baseline and follow up of 0.5, and 10% dropout (we expect less than 10% dropout at three months). With 50 participants, who receive occupational medical assistance, and 50 who do not, a difference in mean *sickness absence percentage* of 11.9% can be detected with power = 0.8, alpha = 0.05, an assumed standard deviation of 20%, and up to 10% dropout at three months; the same power is expected for the *transfer income percentage* after 12 and 13–24 months since these outcome measures are based on register information with practically complete follow-up. Power calculations were carried out with STATA 13 software (StataCorp LP, College Station, TX, USA; power pairedmeans and twomeans, respectively).

According to analyses of data from the Danish National Patient Register and the Danish National Register on Public Transfer Payments, around 1150 patients had an arthroscopic subacromial decompression for SIS in Central Region Denmark in 2008, and 67% of these patients had surgery at public hospitals. Thus, we find it realistic to recruit 130 patients within the inclusion period. Originally, we intended to include 400 patients, but with the change of inclusion criteria and outcome measures for the occupational intervention, the necessary sample size was reduced.

### Economic evaluation

If the results indicate beneficial effects of any of the interventions, costs and effects will be evaluated and compared from a societal perspective after 12 months, using incremental cost-effectiveness ratios, which will be calculated as the ratio of the change in costs to the incremental effects of the interventions in terms of the primary outcome measures. Additionally, we may use quality-adjusted life-years (QALYs), using the authorised Danish version of the EQ-5D-3L health-related quality of life instrument [38]. Direct total costs of the interventions and usual care in the primary and secondary health care sector will be included, together with direct total costs for patients and their employers. Indirect costs, i.e. costs of health-related productivity loss for the employed subgroup (work-time missed and reduced on-the-job effectiveness) due to health problems, will be assessed by the Work Productivity and Activity Impairment Questionnaire - General Health [39], and the value of the patients’ salaries. Intangible costs will not be assessed separately.

### Trial status

Inclusion of patients without paid work for at least 25 hours per week was terminated by 31 December 2013 (n = 126), and data analysis to evaluate the physiotherapy intervention at 3 months is currently ongoing. Recruitment of patients with paid work for at least 25 hours per week will continue until 30 June 2014.

## Discussion

The few studies that have compared the effect of different postoperative exercise programmes have focussed on the initial 4–12 weeks and have targeted all patients after decompression surgery for SIS [13-16]. We chose to start our interventions 8–12 weeks after surgery, where the six orthopaedic departments that contribute patients to SIP routinely schedule postoperative control, and where we thought that we would be able to target our efforts on the subgroup of patients with continuing shoulder problems and an increased likelihood of chronic disability. Any problems regarding work retention would also be crystallised at this time, where unrestricted activities are usually allowed.

The RCT is embedded in a cohort study, which enables evaluation of selection into the project and reach of the intended target group. It is impossible to blind participants and providers of the interventions. To minimise information bias, we included register-based variables and Constant Score as outcome measures and aimed to ensure blinding of the research physiotherapist. Diagnostic practice, preoperative treatment, indications for surgery, and early postoperative training are not standardised, but preoperative treatment and early postoperative training is described by means of questionnaire data. Moreover, preoperative OSS is assessed, which also ensures that comparability with patients from other settings can be judged.

Results of SIP can be incorporated into clinical practice guidelines for rehabilitation after arthroscopic decompression for SIS. If the interventions are successful, information materials will be prepared for use in hospital departments, primary health care, job centres, and training centres, including job-title-based red-yellow-green charts to guide health care professionals and social workers regarding the need for occupational medical assistance. SIP evaluates a new rehabilitation approach, where physiotherapy and occupational interventions are provided in continuity of surgical episodes of care. If successful, the project may serve as a model for rehabilitation of surgical shoulder patients.

## Abbreviations

EQ-5D-3L: Euroqol-5 dimensions-3 levels; JEM: Job exposure matrix; OSS: Oxford Shoulder Score; QALYs: Quality-adjusted life years; RCT: Randomised controlled trial; SIP: Shoulder Intervention Project; SIS: Subacromial impingement syndrome.

## Competing interests

The authors declare that they have no competing interests.

## Authors’ contributions

PF and SWS conceived the project. All authors were involved in the development of the study design. SWS drafted the manuscript in close collaboration with PF. All authors revised the manuscript for important intellectual content and approved the final version.

## Pre-publication history

The pre-publication history for this paper can be accessed here:

http://www.biomedcentral.com/1471-2474/15/215/prepub
